# Individualised multiplexed circulating tumour DNA assays for monitoring of tumour presence in patients after colorectal cancer surgery

**DOI:** 10.1038/srep40737

**Published:** 2017-01-19

**Authors:** Sarah B. Ng, Clarinda Chua, Matthew Ng, Anna Gan, Polly SY Poon, Melissa Teo, Cherylin Fu, Wei Qiang Leow, Kiat Hon Lim, Alexander Chung, Si-Lin Koo, Su Pin Choo, Danliang Ho, Steve Rozen, Patrick Tan, Mark Wong, William F. Burkholder, Iain Beehuat Tan

**Affiliations:** 1 Microfluidics Systems Biology, Institute of Molecular and Cell Biology, Singapore; 2 Division of Medical Oncology, National Cancer Centre Singapore, Singapore; 3 Genome Institute of Singapore, Singapore; 4 Division of Surgical Oncology, National Cancer Centre Singapore, Singapore; 5 Department of Colorectal Surgery, Singapore General Hospital, Singapore; 6 Department of Pathology Anatomical Pathology, Singapore General Hospital, Singapore; 7 Department of Hepatopancreatobiliary/Transplant Surgery, Singapore General Hospital, Singapore; 8 Cancer & Stem Cell Biology Program, Duke-NUS Medical School, Singapore; 9 Cancer Science Institute, National University of Singapore, Singapore

## Abstract

Circulating tumour DNA (ctDNA) has the potential to be a specific biomarker for the monitoring of tumours in patients with colorectal cancer (CRC). Here, our aim was to develop a personalised surveillance strategy to monitor the clinical course of CRC after surgery. We developed patient-specific ctDNA assays based on multiplexed detection of somatic mutations identified from patient primary tumours, and applied them to detect ctDNA in 44 CRC patients, analysing a total of 260 plasma samples. We found that ctDNA detection correlated with clinical events – it is detectable in pre-operative but not post-operative plasma, and also in patients with recurrent CRC. We also detected ctDNA in 11 out of 15 cases at or before clinical or radiological recurrence of CRC, indicating the potential of our assay for early detection of metastasis. We further present data from a patient with multiple primary cancers to demonstrate the specificity of our assays to distinguish between CRC recurrence and a second primary cancer. Our approach can complement current methods for surveillance of CRC by adding an individualised biological component, allowing us not only to point to the presence of residual or recurrent disease, but also attribute it to the original cancer.

Colorectal cancer (CRC) is the third most common cancer worldwide, accounting for 10% of global cancer burden. Many CRC patients undergo curative-intent surgery to remove all gross visible sites of disease – namely the primary tumour and regional lymph nodes for patients with early stage disease, as well as visible metastatic sites for patients with limited volume metastatic disease. After surgery, patients remain at risk of recurrence, and major oncology clinical practice guidelines advocate a structured surveillance program to monitor for recurrence^1^. This involves blood tests for carcinoembryonic antigen (CEA) and periodic CT scans. Current surveillance strategies have limitations. Radiation exposure, costs, logistics and operational constraints limit the frequency at which CT scans can be performed, and CT scans can only reliably detect lesions larger than 1 cm in diameter. Serum CEA can be assayed more frequently but is limited by low sensitivity. Importantly, neither test is completely specific for CRC recurrence and false positive results may be due to conditions with different clinical implications, prognosis and treatment. Elevation of CEA or new lesions apparent on serial CT scans are commonly due to non-neoplastic causes (e.g. infection) or from a different cancer^2^. Thus, a more specific and sensitive biomarker is needed to detect the recurrence of CRC.

Circulating tumour DNA (ctDNA) has emerged as a potential tumour biomarker and liquid biopsy across cancers^3^, and is typically detected through assays for somatic tumour mutations in DNA from patient plasma. Detection of ctDNA has the advantage of being highly specific, because such mutations should occur from tumour cells. In CRC, the potential of ctDNA for monitoring disease has been shown in the metastatic setting, particularly in detecting resistance to anti-EGFR therapy^3^,^4^,^5^, response to chemotherapy^6^ and residual disease post-surgery^7^. We thus sought to develop an assay that could detect ctDNA in relation to various clinical events attributable to a patient’s CRC, in particular to determine if detection of metastatic recurrence earlier than conventional surveillance post-primary resection was possible. At the onset of our study, the latter had not been shown, but more recent reports have indicated the potential to do so in limited cohorts^8^,^9^.

One issue in developing ctDNA assays is determining which and how many somatic mutations to assay for. Most of the plasma samples in these previous studies were assayed only for a single mutation; in cases where a few mutations were assayed, each was a separate assay requiring independent sample aliquots. In both instances, this could limit the sensitivity of detection, particularly in the case of ctDNA where input amounts and mutation frequency are low. Simultaneous detection of multiple somatic mutations would be ideal, but finding multiple hotspot mutations that all patients harbour is not possible and personalised assays based on patient-specific mutations would be required.

It was initially uncertain if somatic mutations from patient primary tumours resected up to three years prior would still be relevant for detection in plasma at and after the development of metastasis. Previous studies that used tumour tissue to determine mutations to monitor in ctDNA used tissue fairly contemporary to the plasma assayed. However, we and others showed that there is a large degree of genomic concordance amongst somatic mutations (about 80%) in each patient’s primary tumour and corresponding metastatic lesion^10^,^11^. This suggested that we could indeed use somatic mutations identified from a patient’s surgical primary CRC specimen to provide an individualised “nucleic acid thumbprint” for detection in plasma at metastasis, specific not only to the patient, but also to the particular cancer.

Various methods for accurately detecting ctDNA at low fractions have been described, including digital PCR^8^,^12^, sequencing of PCR amplicons^13^,^14^,^15^,^16^ or sequencing of hybridisation captured libraries^17^,^18^. Digital PCR is highly sensitive, but requires allele-specific primers or probes for each somatic mutation assayed and does not multiplex easily. In comparison, using sequencing as a read-out enables the detection of multiple mutations simultaneously, whether using PCR or capture as an enrichment step. However, to detect low frequency variants with a sequencing readout, it is necessary to correct for sequencing noise as well as errors introduced during library amplification.

To address these issues, we developed an approach to detect ctDNA based on multiplex-PCR amplicon sequencing of patient-specific primary somatic mutations in plasma (Fig. 1), including an error correction method for detection of low-frequency mutations in sequencing data that is generalizable for personalised assays. To determine the applicability of this approach, we also analysed plasma samples from 44 patients with CRC, in three subsets: A) in pre- and post-operative plasma samples to evaluate whether our detection of ctDNA corresponds to tumour presence, B) in post-recurrence samples to determine if ctDNA is detectable in patients with recurrent disease and C) in pre-recurrence samples to evaluate the sensitivity of ctDNA for early detection of CRC recurrence. We also present results from a patient who had undergone surgery for metastatic colorectal cancer who developed a new liver lesion on CT scan that was due to a different cancer (cholangiocarcinoma) which was distinguished non-invasively by an individualised ctDNA assay.

## Results

### Development of multiplex amplicon sequencing assays based on patient primary-tumour-specific (PPS) somatic mutations

We called somatic variants in each of our 44 patients using targeted hybridisation capture and shotgun re-sequencing of matched primary tumour and normal tissue samples, finding a median of 20 somatic variants (range: 8–2245) per patient. One patient had a DNA polymerase ε mutation leading to an “ultramutation” phenotype. There was minimal overlap of mutations between patients, with at most one shared mutation between any two pairs (Supplementary Fig. S1). KRAS G12V and KRAS G12D were the most frequently observed mutations, found in six samples each. For each patient, we designed multiplex PCR assays for amplicon sequencing to amplify these patient primary-tumour-specific (PPS) variants, averaging 10 (range: 4 to 14) amplicons per patient after removal of poorly performing amplicons and primers contributing to dimers. Re-sequencing patient tumour tissue samples with these multiplex PPS assays verified the presence of nearly all of the somatic mutations (402 of 407 amplicons with at least 100X read depth), and the tumour mutation allele fraction estimated from the PPS multiplex amplicon sequencing assays was concordant with the estimates from the initial target-capture sequencing (Pearson: r^2^ = 0.88, Supplementary Fig. S2).

### Development of variant calling pipeline

**Figure 2:**
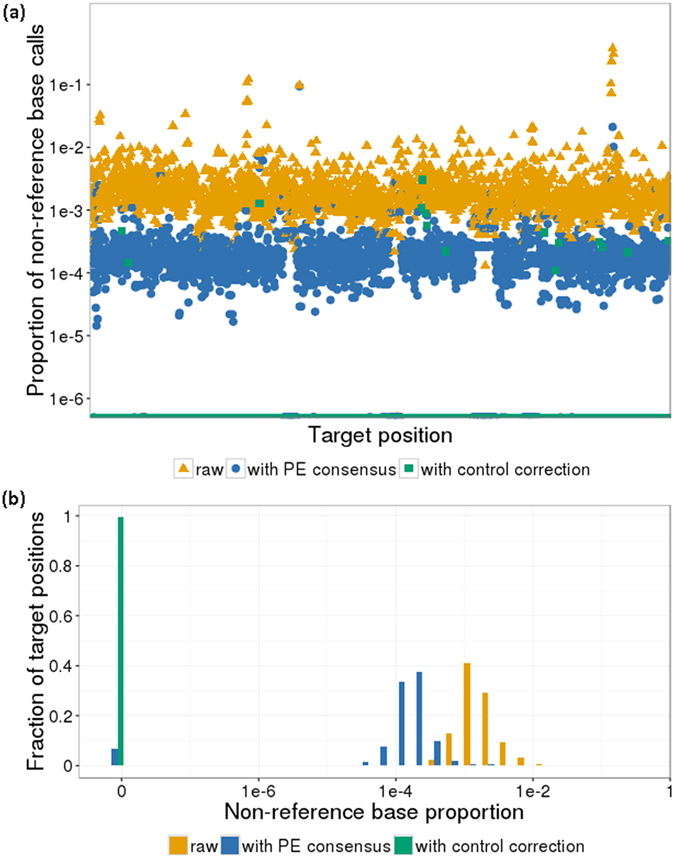
Error correction reduces non-reference base calls in negative (healthy) samples. (**a**) Using paired-end consensus calling (blue, circles) and removal of noise as modelled in control samples (green, squares), the non-reference base call proportion is reduced from raw calls (orange, triangles). Points are plotted as the average of 3 samples over each target position (2619 bp after sites of common variation were removed), with zero-value points at the bottom. (**b**) Histogram representation of the data in (**a**).

In order to sensitively detect low frequency ctDNA mutations and distinguish them from noise, we developed a variant calling pipeline with low base-calling error rates. We first applied six different PPS assays on plasma from three healthy individuals (Fig. 2). Using raw sequencing reads, we observed an average of 2.8 × 10^−3^ non-reference calls per base after removing sites of common population variants. We then used a paired-end read merger^19^ to determine consensus bases for read pairs that had overlapping positions and remove discordant calls, which reduced the non-reference base call rate by 10-fold to 2.6 × 10^−4^. We did further error correction by removing calls if they occurred at a low proportion indistinguishable from an estimate of noise: specifically, if they occurred at a proportion not significantly higher than the maximum proportion of non-reference bases observed in three same-run negative controls (Hapmap cell-line DNA) as determined by an exact Poisson test. We found that this reduced the average non-reference calls to 3.7 × 10^−6^ per base, with 99.35% of bases having no errors. This pipeline was used for the rest of our variant calling.

### PPS assays are highly sensitive and specific for ctDNA

**Figure 3:**
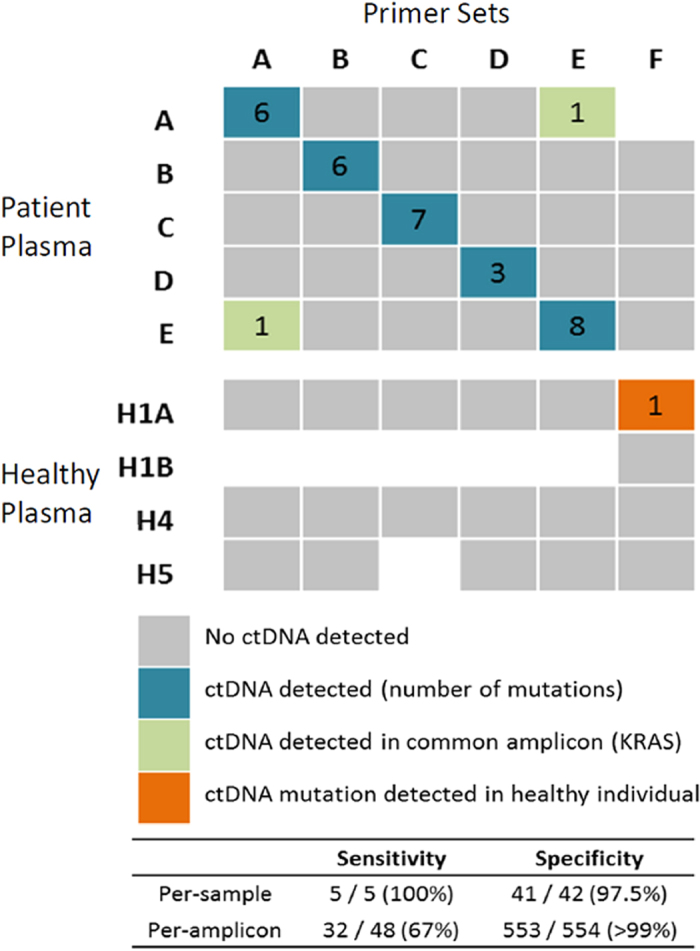
Sensitivity and specificity of patient-specific amplicon-sequencing assays for detection of ctDNA in plasma. Plasma from 6 patients (labelled **A–E**) and 3 healthy volunteers (H1,H4,H5) were assayed for ctDNA using 6 different patient-specific primer sets (labelled **A–F**). Primer set labels match that of the patient they were designed for. Plasma from volunteer H1 was taken from a later timepoint (H1B) and re-tested with the same primer set F. This sample H1B was not used for calculations of sensitivity and specificity.

We estimated the sensitivity and specificity of the PPS assays using the data from the healthy individuals in combination with data from the same six assays tested on plasma from five patients (Fig. 3, Supplementary Fig. S3). Mutations were detected in all five samples where the plasma was from the same patient that the amplicon set was designed for (sensitivity of 100%). In addition, in the two instances where two patients had a KRAS mutation in common, only the KRAS mutation was detected. In all other patient plasma where the amplicon sets did not match the expected variants, no tumour variants were detected (n = 22), and in healthy individuals (n = 11), only one tumour variant was detected, for a per-sample specificity of 97.5%. This variant was not detected on replication of this assay in plasma from another timepoint from the same healthy individual. Looking at the amplicons in aggregate, 32 out of 48 variants expected to be present in plasma samples were detected (sensitivity 67%), whereas only one out of 554 expected negative amplicons was called positive (specificity >99%). This highlights the utility of assaying for multiple mutations per sample – although there was not complete sensitivity on a per-amplicon basis, but overall there was increased likelihood that at least one amplicon per positive sample yielded a positive variant call.

### PPS assays are quantitative and can detect low-frequency variants down to 0.05%

**Figure 4:**
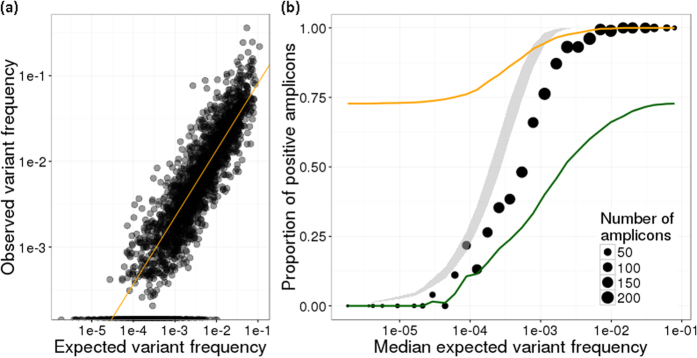
Multiplex amplicon assays performed on serial dilutions of tumour DNA exhibit a linear correlation between observed and input mutation frequencies and can sensitively detect low-frequency variants. (**a**) The expected vs observed variant frequency for each amplicon in the dilution series is shown (Pearson: r^2^ = 0.89) with the fitted linear model in orange. (**b**) Amplicons were binned by expected variant frequency and the proportion of positive amplicons (i.e. input mutation detected) is shown. The number of amplicons in the bin is indicated by size of the dot, and the grey ribbon indicates the expected proportion of positive amplicons in 3000 genomic equivalents based on the minimum and maximum of the expected frequencies in the bin under a binomial distribution. The cumulative proportion of positive amplicons at the expected variant frequency and higher (orange line) and lower (green line) are also plotted.

To ascertain if the mutation frequency estimated by each amplicon assay was linearly correlated with the input, as well as determine the sensitivity of the assays for low frequency mutations, we performed amplicon sequencing on serial dilutions of tumour DNA such that the expected mutation allele frequency over all amplicons had a range of 0.0002–1%. After filtering for a minimum read depth of 1000X across the whole dilution series, 323 amplicons remained, of which 70% had a correlation coefficient of >0.95 between the expected and observed variant frequencies (Supplementary Fig. S4), indicating a good linearity over at least three orders of magnitude. In general, variants were detected in 95% of amplicons with an expected variant frequency of 0.1% or higher, and in 24.9% of amplicons with expected variant frequency lower than 0.05% (Fig. 4).

### PPS-ctDNA is detectable in patient plasma samples

Using the PPS assays, we analysed a total of 260 plasma samples from 44 patients with colorectal cancer to see if we could detect patient primary tumour mutations in matched patient plasma. This would indicate the presence of ctDNA. During analysis, it was discovered that one series of plasma samples was attributed to the wrong patient, leading to assaying of the wrong somatic mutations in plasma. Data from this series (n = 4) was removed from further results. 149 plasma samples of the remaining (58%) were positive for at least one PPS mutation and 38 patients had at least one positive sample. We henceforth refer to the detection of patient-specific tumour mutations in plasma as PPS-ctDNA. Of the plasma samples that corresponded to the clinical presence of disease (pre-operative or post-recurrence, n = 187), 131 had PPS-ctDNA detected (70%). The median duration of time from primary surgery to the first and last available post-operative sample was 371 and 864 days respectively, with the median duration of follow-up from the first and last available post-operative plasma samples being 965 and 849 days (range of 786–1253 and 91–1226).

### PPS-ctDNA is detectable in pre-operative but not post-operative plasma

To determine if PPS-ctDNA is specific for tumour presence in patients, we analysed a set of plasma samples from 13 patients, taken immediately before (i.e. tumour present) and within five days after the patient underwent surgery (i.e. tumour absent) for removal of primary tumour (Table 1). 12 of these patients had early stage disease and had curative-intent surgery to remove all visible disease including regional lymph nodes. Of these patients, 10 had detectable PPS-ctDNA (frequency range: 0.06–1.07%) pre-operatively and of the 10 available samples post-operatively, none had detectable PPS-ctDNA. The remaining patient underwent palliative resection of the primary tumour in the presence of metastatic disease, and this is reflected in both the pre- and post-operative plasma being positive for PPS-ctDNA. Together, these results indicate that PPS-ctDNA is specific for tumour presence.

### PPS-ctDNA is detectable in patients with recurrent CRC

We sought to determine if PPS-ctDNA is detectable post-recurrence despite a potentially long period of time between the resection of the primary tumour, from which the PPS assay was designed, and the emergence of the metastatic tumour, which is presently contributing ctDNA. To this end, we analysed 159 plasma samples taken from 26 patients after recurrence had been detected. 25 of these patients (96%) had detectable PPS-ctDNA, providing support that because of sufficient genomic similarity between primary and metastatic disease, the same mutations present in a patient’s primary tumour can later be detected in the plasma when recurrent CRC develops.

**Figure 5:**
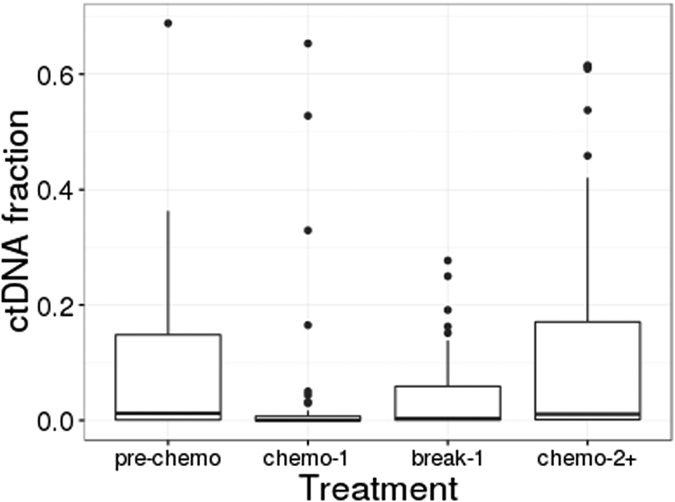
ctDNA fraction detected in metastatic patients across the course of chemotherapy. Boxplots of the ctDNA fraction in plasma samples obtained from patients after recurrence and during treatment with chemotherapy is shown in the following subsets: treatment naïve (“pre-chemo”), during first-line chemotherapy (“chemo-1”), during first-line chemotherapy break (“break-1”), and during all further lines of chemotherapy or re-challenges (“chemo-2+”). These subsets reflect the change in patient treatment over time.

In general, we also observe that the level of PPS-ctDNA varies with treatment over time (Fig. 5). Overall, PPS-ctDNA was detectable in 110 of 159 post-recurrence plasma samples (69%). Prior to treatment with chemotherapy, PPS-ctDNA is detectable in 15 out of 19 samples (79%). During first-line chemotherapy, detection of ctDNA drops (22 of 51 samples; 43%) as well as the fraction of ctDNA detected, corresponding to patient response to treatment. Post-chemotherapy, ctDNA becomes detectable again (19 of 25 samples; 76%) due to progression of disease, which further lines of chemotherapy are unable to forestall (detection in 53 of 61 samples; 86%).

### PPS-ctDNA is detectable in patient plasma before clinical diagnosis of recurrence

**Figure 6:**
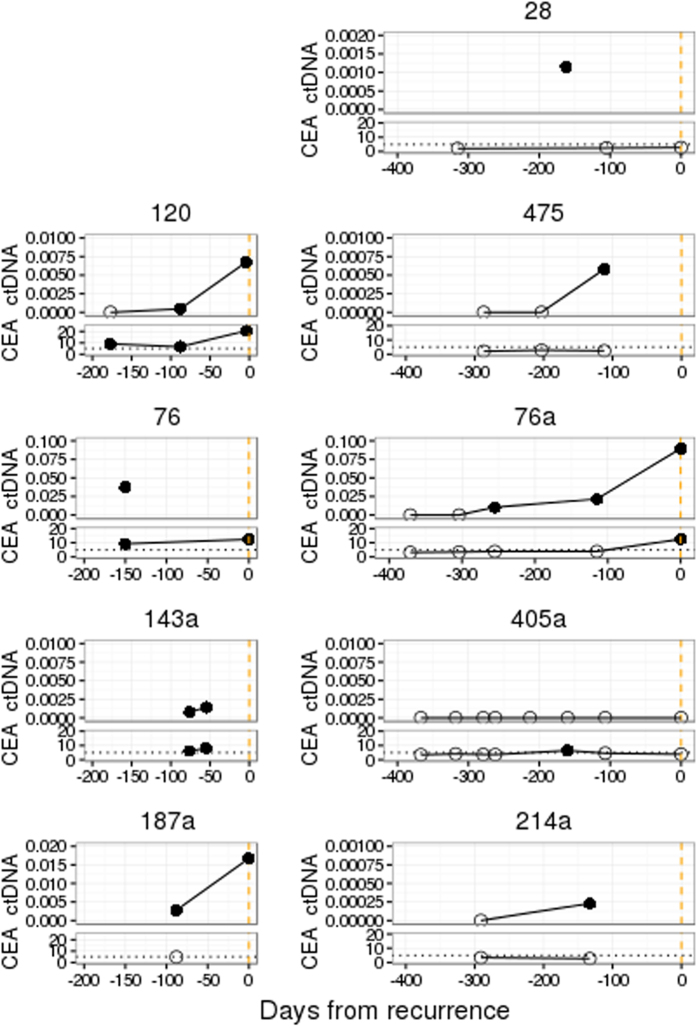
ctDNA fraction and CEA levels in patients with plasma from before recurrence was diagnosed. Only patients with multiple timepoints are shown (9 of 15 events). Events which occurred in patients post-metastectomy are identified with an “a”. Filled circles refer to detection of ctDNA (>0) or elevated CEA (>5 ng/ml). Orange dashed line indicates day of clinical detection of recurrence.

To determine if PPS-ctDNA is detectable before clinical recurrence, we analysed 37 plasma samples that were collected prior to or at 15 diagnoses of clinical recurrence after surgery with curative intent (ten primary resections and five metastectomies). This corresponded to 13 patients as two patients suffered a re-recurrence after metastectomy. PPS-ctDNA was detected in 11 out of 15 cases with a lead time of up to 255 days (Fig. 6). Of the 34 plasma samples analysed, 32 had coincident measurement of CEA. There was no case with sustained elevated CEA levels but no detection of ctDNA, and three cases of detectable ctDNA without a corresponding CEA rise.

### Detection of PPS-ctDNA in the absence of recurrent CRC

In our cohort, we had a total 12 patients with post-operative (curative primary resection or metastectomy) plasma samples who were recurrence-free at follow-up. The majority (n = 10) have no detectable PPS-ctDNA, concordant with their clinical status as recurrence-free at a median of 877 (range: 124 to 946) days of follow-up since the last negative post-op sample which was obtained. One patient had detectable PPS-ctDNA one month post-surgery, which subsequently became undetectable after post-operative chemotherapy. This patient remains recurrence free 880 and 849 days after the first positive and the last negative timepoint. Another patient had detectable PPS-ctDNA in three timepoints spanning 707 days, starting 129 days after surgery, but remains recurrence-free, 818 and 111 days after the first and last positive timepoint respectively.

### PPS-ctDNA can distinguish between metastasis and a different cancer

A different cancer (second primary) may develop in patients with a previous history of CRC. This may manifest as a radiological mass on the CT scan together with elevation of non-specific tumour markers (e.g. CEA). To demonstrate as a proof-of-concept that PPS-ctDNA can distinguish this situation from a metastatic case, we identified a patient who had undergone synchronous surgery of the primary CRC and liver metastasis, then subsequently developed a liver mass and elevation of CEA that was initially thought to be due to recurrent CRC, but later revealed to be a cholangiocarcinoma on histopathology upon surgical resection.

**Figure 7:**
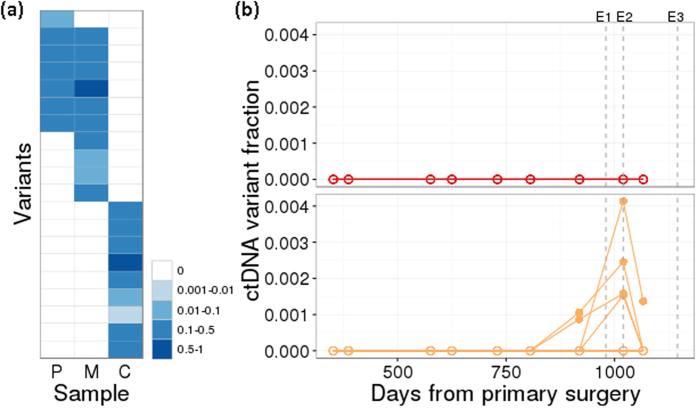
Detection of ctDNA variants is highly specific to tumour origin. (**a**) Variant frequency of 20 somatic tumour variants identified by targeted capture sequencing (upper 11 specific to the original CRC primary [P] and liver metastasis [M]; lower 9 specific to second cholangiocarcinoma [C]) as assayed by a patient-specific multiplex amplicon assay in tumour tissue DNA. (**b**) ctDNA variant fraction over multiple timepoints post-primary surgery. The 11 CRC primary/metastasis variants (upper panel, red) and 9 cholangiocarcinoma variants (lower, orange) are plotted separately. Each line refers to a separate variant. E1: detection of liver lesion. E2: surgery to remove second primary. E3: clinical detection of a lung metastasis by CT scan.

From this patient, we sequenced the primary tumour and liver metastases, as well as the cholangiocarcinoma, and developed a multiplex assay based on mutations from all three organ sites. This assay was validated on tumour tissue DNA (Fig. 7a), and then applied to plasma samples collected from multiple timepoint between the patient’s two surgeries, as well as after the cholangiocarcinoma surgery (Fig. 7b). We observed that mutations present in the patient’s primary/liver metastases were absent from the plasma, consistent with the absence of recurrent CRC. Instead, mutations corresponding to the cholangiocarcinoma were detected, up to 2 months before clinical detection of the liver lesion. After resection of the cholangiocarcinoma, one mutation corresponding to the cholangiocarcinoma was also present 79 days prior to the patient developing subsequent lung metastases, revealing residual disease post resection of the cholangiocarcinoma prior to overt lung metastases becoming clinically evident as well as providing molecular evidence that this last recurrence was attributable to the cholangiocarcinoma and not the previous CRC.

## Discussion

The goal of our study was to develop a personalised surveillance strategy to monitor the clinical course of colorectal cancer after cancer surgery. In particular, we wanted to be able to simultaneously assay for the presence of multiple somatic mutations identified from the CRC primary tumour in patient plasma and determine the correlation with clinical events. In CRC, because of the high degree of genomic similarity of the primary and metastases^10^,^11^, the resected surgical specimen provides an available mutational profile to monitor and or provide diagnostic support for the recurrence of the same cancer. Similar studies have recently shown that ctDNA can be detected in CRC patients post-surgery. In particular, a retrospective study of ten patients, half of which had CRC recurrence, showed that personalised digital droplet PCR (ddPCR) assays against structural variants identified from tumour whole genome sequencing could be used to detect recurrence early^8^. Also, in a prospective study of only stage II patients using the Safe-Seq assay against single somatic mutations, they showed that detection of ctDNA at a single time-point four weeks after surgery predicted a high risk of recurrence^9^. Here, we sought to describe a more general assay based on standard PCR techniques and sequencing to detect multiple somatic mutations per patient, and its general applicability across various CRC patients at differing stages with plasma samples obtained at varied time points after initial primary surgery.

Using patient-specific somatic mutations, we were able to design a personalised ctDNA detection assay for each of the patients in our study. The primary challenge in using massively parallel sequencing as a read-out for mutation detection in amplicons is the need to do error-correction for artefacts arising from PCR or sequencing. One error-correction method is the use of unique molecular identifiers to tag input molecules and thus enable consensus calling to increase confidence in base calls^13^,^17^,^18^, but this inflates the total amount of sequencing required, with one estimate at 40 times the read depth desired^20^. This quickly becomes infeasible considering the low frequencies at which ctDNA mutations generally occur. Another error-correction approach is the use of a large number of control samples to develop per-base error models to remove false positive calls^14^,^16^. This works well for standardised panels, but becomes onerous for individualised assays. Here, using only three controls per sample, we were able to develop a robust error-correction approach even for personalised assays. Our approach has the added advantage that the controls can be processed simultaneously with samples, avoiding potential batch effects. We chose to model the read counts using the Poisson distribution because it is discrete, and more robust to situations where there are low (or no) observed variant reads in the controls or varying read depth, as compared to using a test of two proportions. In practice, using a one-tailed test that the proportion of variant reads in sample is more than that of the controls would give similar results (data not shown). An alternative read count model uses the negative binomial distribution^16^, but three controls are insufficient to estimate the model parameters, so we have instead used the read counts from the control with the maximum variant proportion in order to correct for potential overdispersion.

It is also necessary to consider that tumours may have different genomic profiles over space (intra-tumoural heterogeneity) and time (between primary and metastatic lesions). This could affect our ability to reliably identify somatic mutations, initially found in one random sector of a primary tumour, in plasma ctDNA from the patient at a later time. To mitigate this potential problem, we chose to develop assays against multiple mutations per patient, averaging 10 mutations instead of just one or two. This gives us multiple chances to assay for ctDNA, even if mutations may be missing due to heterogeneity. Furthermore, based on recent studies we do not expect that tumour heterogeneity to be a major issue. As already noted above, it has been shown that, at least in CRC, there is about 80% concordance between somatic mutations found in primary and metastatic tumours^10^,^11^. Also, recent studies by us – using targeted capture sequencing with the same 799-gene panel used here – as well as others, have further demonstrated that there is a high level of concordance of mutations present across different sectors of patients’ primary colorectal tumours^21^,^22^,^23^. As such, tumour heterogeneity should not significantly affect our assays.

In over 320 designed amplicons, the majority of our assays had an acceptable linearity of detection of mutant fraction over 3 orders of magnitude. This is not as high as previously reported for droplet digital PCR (ddPCR) and amplicon sequencing, and could be attributed to a number of factors, including that we used serial dilutions of tumour DNA with normal tissue DNA instead of PCR products at known molarity^16^ and that we report the presence of ctDNA as a proportion of reads instead of as the total number of mutant genomes present^8^. Nonetheless, our data suggest that our assays are sensitive down to a 0.1% allele frequency per-variant. Furthermore, sensitivity of detection has been shown to increase as the number of variants per sample is assayed^18^. As we assay an average of 10 amplicons per sample, and we observe that 76% of variants around 0.1% (Fig. 3) are detected, we expect that our overall sensitivity to detect ctDNA at 0.1% is >99.99% (1–0.24^10^).

When applied to plasma samples, our assays were able to detect ctDNA in 69% of “post-recurrence” samples where disease was confirmed to be clinically present. The majority of the negative samples were from timepoints when patients were undergoing chemotherapy, and indeed from all patients which we had samples from both pre-treatment and the first timepoint after start of chemotherapy (n = 7), we observed a decrease of ctDNA fraction to undetectable. Correspondingly, we observed increasing ctDNA levels as patients re-recurred after metastectomies or became resistant to chemotherapy and had progressive disease. There was only one patient who was ctDNA negative over all sampled post-recurrence timepoints, and potential reasons for this include the size and location of the metastatic lesion (small, inaccessible to circulation), or possibly that the presumed metastasis represents an independent second tumour that does not have the same somatic mutations as identified in the original primary.

We also observed one instance of persistently elevated PPS-ctDNA in the absence of radiological and clinical disease. This may point to the presence of dormant disease perhaps under some form of immune control. We note that a similar observation was found in a recent study^9^, where three patients were observed to have positive ctDNA post-surgery yet had not recurred by last follow-up, 11, 13, and 19 months after surgery.

Our method is scalable and based upon widely available tools. Somatic variant identification from tumour tissue is becoming increasingly routine and amplicon sequencing does not rely on specialised instruments or reagents other than a high throughput sequencer. However, there is an operational limitation to our approach is that it can be inefficient to develop, store and deploy personalised assays. As we observed in this study, a potential sample mixup would invalidate the test as the wrong mutations would be assayed for. We suggest that this can be overcome by either developing a single large panel assay – which has the disadvantage of requiring much more sequencing, or else a stratification approach could be taken, wherein the majority of patients are served by a small general mutation panel, and personalised or large panels only developed for the remaining minority.

In our cohort, ctDNA could detect recurrence prior to clinical detection and in several cases, in the absence of elevated CEA. We also showed that ctDNA analysis could distinguish between tumours derived from different primary tumours while elevated CEA was non-specific. Detection of ctDNA can complement current surveillance with radiology and non-specific tumour markers by adding an individualised biological component, allowing us to point to the presence of residual/recurrent disease attributable to the original cancer. A larger prospective study is planned to determine the clinical utility of using new molecular blood tests, such as detection of ctDNA, to improve patient outcomes.

## Methods

### Patient recruitment

Patients were recruited in a prospective observational study approved by the institutional review board (Singhealth Centralised IRB- 2013/110/B) where serial plasma specimens were collected from patients with colorectal cancer. All patients provided signed informed consent. Samples were deidentified and all methods performed in accordance with relevant guidelines and regulations. From this study, we selected 45 clinically informative patients (44 for the initial cohort and one with a second primary) where both plasma samples corresponding to clinically informative time-points were collected and archival frozen tissue specimens (tumour and corresponding normal tissue) were available from the Singhealth Tissue Repository. Patient characteristics are given in Supplementary Table S1.

### Somatic variant calling from tumour tissue

DNA extraction, sequencing library preparation, targeted capture and sequencing were performed as previously described^10^. Very briefly, target regions comprised of the coding regions of 799 cancer-associated genes, and somatic variants were identified and annotated from the tumour-normal sequencing after variant calling by either GATK, LoFreq or MuTect.

### Plasma processing

Plasma was obtained from patient blood within 2 hours of venipuncture, followed by centrifugation of blood at 1900 g and 4 °C for 10 min, followed by a second centrifugation of the plasma fraction at 16000 g and 4 °C for 10 min. Plasma was stored at −80 °C until extraction. DNA from plasma was extracted using the QiaAmp Circulating Nucleic Acids Kit (Qiagen), following manufacturer’s instructions.

### Variant selection and primer design

For each patient, up to 15 somatic variants were selected for amplicon design, with priority given to: a) variants at a site amplified by a previously tested primer pair, b) variants in genes associated with colorectal cancer, c) missense variants and short insertions or deletions (indels), d) splice-site variants, e) synonymous variants, f) variants in untranslated regions or introns.

Primers to amplicons containing specified variants were designed using primer3 (version 2.3.4, https://sourceforge.net/projects/primer3/) using default parameters except that a) primer length was allowed to be between 15 and 26 nt, b) primer calculated melting point (Tm) was allowed to be between 50–68 °C (optimum at 59 °C), and c) amplicon lengths were constrained to be short, either between 60 and 100 bp or between 101 and 150 bp. Primer pairs were tested individually and those that did not give the expected product size when analysed by gel electrophoresis were dropped from subsequent use.

### PCR conditions

Individual primer pairs (singleplex) were tested under the following PCR conditions: a 20 ul reaction consisting of 1X Phusion HF buffer, 0.2 mM of each dNTP, 1 uM of each primer, 10 ng of input DNA and 0.02U of Phusion Hot Start II Polymerase (ThermoFisher), with an initial denaturation at 98 °C for 30 sec, 30 cycles of denaturation at 98 °C for 10 sec, annealing at 60 °C for 10 sec and extension at 72 °C for 15 sec, and a final extension at 72 °C for 5 min. Multiplex PCRs were performed under similar conditions, with the only differences being a 40 ul reaction, 0.1 uM of each primer, and an additional 1 mM of MgCl_2_. Further PCR to add sequencing adapters were performed as per singleplex conditions, except without limit on input DNA, and for only 15 cycles.

### Multiplex PCR and amplicon sequencing

Amplicons were generated using multiplex PCR (up to 15 primers per primer set), and products purified using the Agencourt AMPure system (with bead to sample ratio at 1:1, Beckman Coulter) as per manufacturer’s instructions. A second PCR to add on sequencing adapters was then performed, and products similarly purified (1.4:1 bead to sample ratio). Amplicons were then sequenced on the Illumina Miseq (2 × 150 bp) following manufacturer’s instructions. Each sample had up to three same-run negative controls (DNA from Coriell Hapmap CHB cell-line samples) processed together.

### Optimisation of primers for multiplex PCR

In the initial testing of primer pairs in multiplex, reads that were filtered out for being too short (<40 nt) were analysed for primer dimers. Primers contributing to a high number of primer dimers (>20% of sequencing reads) were removed from the multiplex set for subsequent use. Primer sets were also tested on tumour-normal pairs and the variant allele frequency (VAF) of the mutation assessed. Amplicons for which the VAF was 0 in the tumour sample, or for which the VAF was similar (<2-fold) to that in the normal tissue were removed from further consideration.

### Variant detection

Paired-end sequencing reads were pre-processed by PEAR^19^ to remove common adapter sequence and change non-matching bases to N (option –nbase). Reads were then aligned to hs37d5 (http://ftp.1000genomes.ebi.ac.uk/vol1/ftp/technical/reference/phase2_reference_assembly_sequence/hs37d5.fa.gz) using bwa-mem^24^. Aligned reads were converted to extended CIGAR representation including mismatches (https://samtools.github.io/hts-specs/SAMv1.pdf). For each amplicon, read counts for reference reads and reads corresponding to the mutation were extracted. Amplicons were considered not-called if an initial read-depth filter was not met (1000X). A one-tailed exact conditional test of the ratio of two Poisson rates^25^ was used to determine if the number of reads containing the expected variant was significantly more than that from the control sample with the highest proportion of the corresponding read.

### Determine significance cut-off for exact test

In order to determine an appropriate level of significance for the exact conditional test, we constructed a receiver-operator characteristic (ROC) curve based on expected positive amplicons from patient plasma samples as well as expected negative amplicons from plasma from healthy individuals as well as non-matching patient plasma samples (Supplementary Fig. 3). We then chose a significance (p-value) cut-off (0.01) between the point with maximum accuracy (p-value = 0.004) and the next point (p-value = 0.013).

### Tumour dilutions

Linearity and sensitivity of the assay was assessed using serially diluted tumour DNA, where tumour DNA was mixed with corresponding normal DNA at the following ratios: 1:10, 1:20, 1:40, 1:80, 1:160, 1:320, 1:640, 1:1280 and 1:2560. The expected variant frequency was calculated from the variant frequencies as observed in tumour and normal tissue samples and compared to the observed variant frequencies.

## Additional Information

**How to cite this article**: Ng, S. B. *et al*. Individualised multiplexed circulating tumour DNA assays for monitoring of tumour presence in patients after colorectal cancer surgery. *Sci. Rep.* **7**, 40737; doi: 10.1038/srep40737 (2017).

**Publisher's note:** Springer Nature remains neutral with regard to jurisdictional claims in published maps and institutional affiliations.

## Supplementary Material

Supplementary Information

## Figures and Tables

**Figure 1:**
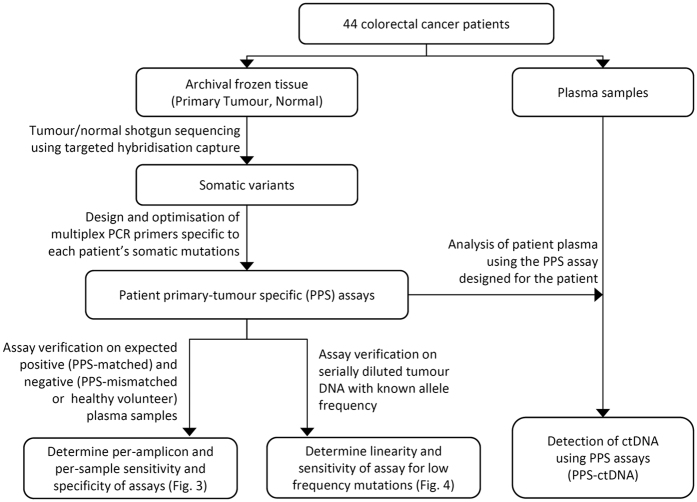
Workflow for design and use of patient primary-tumour specific (PPS) assays to detect circulating DNA (ctDNA).

**Table 1:** Detection of ctDNA in plasma samples obtained from patients just before surgery to remove primary tumour (“pre-op”) and within five days after surgery (“post-op”).

Patient	ctDNA fraction		Days after surgery
	Pre-op	Post-op	
*Early stage cancer, curative-intent surgery*			
130	0.0022	ND	3
206	0.0006	ND	4
405	0.0047	ND	4
410	0.0011	—	—
417	ND	ND	5
440	0.0107	ND	4
441	ND	ND	4
442	0.0019	—	—
478	0.0050	ND	3
480	0.0015	ND	4
486	0.0065	ND	4
495	0.0038	ND	4
*Metastatic cancer, palliative resection*			
494	0.0135	0.021	3

‘ND’ refers to ‘not detected’ and a dash means no plasma sample was available.
