# Supplementary material for: Individualised multiplexed circulating tumour DNA assays for monitoring of tumour presence in patients after colorectal cancer surgery
Source: Sci Rep. 2017 Jan 19;7:40737. doi: 10.1038/srep40737 (PMC5244357; doi:10.1038/srep40737)
Supplement: Supplementary Information [file srep40737-s1.pdf]

SUPPLEMENTARY INFORMATION

for

Individualised multiplexed circulating tumour DNA assays for monitoring of tumour presence in patients after colorectal cancer surgery.

Sarah B Ng^*[1], Clarinda Chua[2], Matthew Ng[2], Anna Gan[3], Polly SY Poon[1], Melissa Teo[4], Cherylin Fu[5], Leow Wei Qiang[6], Lim Kiat Hon[6], Alexander Chung[7], Si-Lin Koo[2], Su Pin Choo[2], Danliang Ho[2], Steve Rozen[8], Patrick Tan[8,9], Mark Wong[5], William F Burkholder[1], Iain Beehuat Tan*[2,3,,8]

[1]Microfluidics Systems Biology, Institute of Molecular and Cell Biology, Singapore
[2]Division of Medical Oncology, National Cancer Centre Singapore, Singapore
[3]Genome Institute of Singapore, Singapore
[4]Division of Surgical Oncology, National Cancer Centre Singapore, Singapore
[5]Department of Colorectal Surgery, [6]Department of Pathology, [7]Department of Hepatopancreatobiliary / Transplant Surgery, Singapore General Hospital, Singapore
[8]Cancer & Stem Cell Biology Program, Duke-NUS Medical School, Singapore
[9]Cancer Science Institute, National University of Singapore, Singapore

^Current affiliation: Genome Institute of Singapore, Singapore
*Correspondence to: Sarah Ng (ngbhs@gis.a-star.edu.sg) and Iain Tan (iain.tan.b.h@singhealth.com.sg)

Supplementary Figures S1-S4
Supplementary Table S1

Supplementary Figure S1: Number of somatic mutations identified in 44 patient tumours.

The number of mutations in each sample is given on the diagonal (range: 8 – 2245), and as well as the number of mutations any pairwise samples share (lightly shaded boxes, maximum is 1). Mutations which are shared among at least three samples are specifically identified (A-F).

404	18	7	30	38	120	198	81	31	429	478	28	214	29	484	373	439	153	410	68	206	51	480	188	76	405	486	494	187	260	183	315	382	398	442	417	440	143	475	495	61	441	49	130	
8																																				1								404
	9	D								A	A	A						A	A					D																				18
		9	B					B					B				B							D																		B		7
			9					B					B				B													1												B		30
				10																																		1						38
					11																																							120
						11																																						198
							13																																					81
								15					B				B																									B		31
									15											E							E																	429
										16	A	A						A	A																									478
											16	A						A	A																									28
												16						A	A				C	C														C						214
													16				B																			1						B		29
														16																							1							484
															16						1								1															373
																17															1													439
																	18																						1			B		153
																		19	A											1														410
																			19																									68
																				19							E	F	F															206
																					19				1																			51
																						19																						480
																							20	C														C						188
																								20														C				1		76
																									20														1					405
																										20				1														486
																											21																	494
																												21	F															187
																													21													1		260
																														21														183
																															22													315
																																22												382
																																	24	1										398
																																		25										442
																																			27							1		417
																																				27								440
																																					28							143
																																						28						475
																																							31					495
																																								31				61
																																									41			441
																																										112	1	49
																																											2245	130

	Gene	Mutation	Samples with mutation
**A**	KRAS	G12V	6
**B**	KRAS	G13D	6
**C**	TP53	R273H	4
**D**	APC	R232X	3
**E**	TP53	R273C	3
**F**	APC	R876X	3

Supplementary Figure S2: Concordance of somatic mutation allele frequency as estimated by targeted sequencing and amplicon sequencing

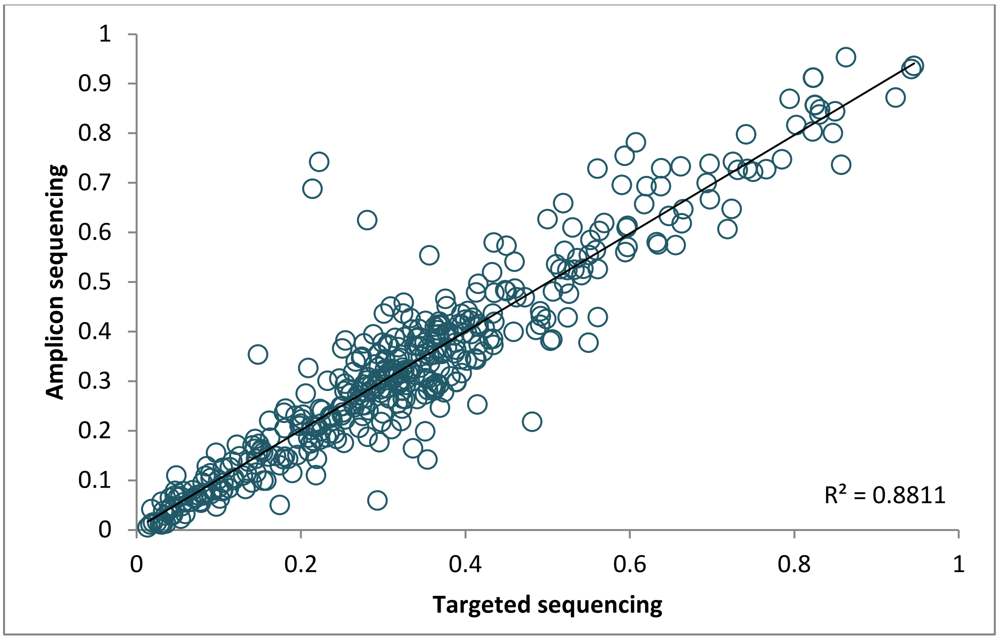

Supplementary Figure S3: ROC curve of positive and negative amplicons based on plasma samples from patients and healthy volunteers respectively.

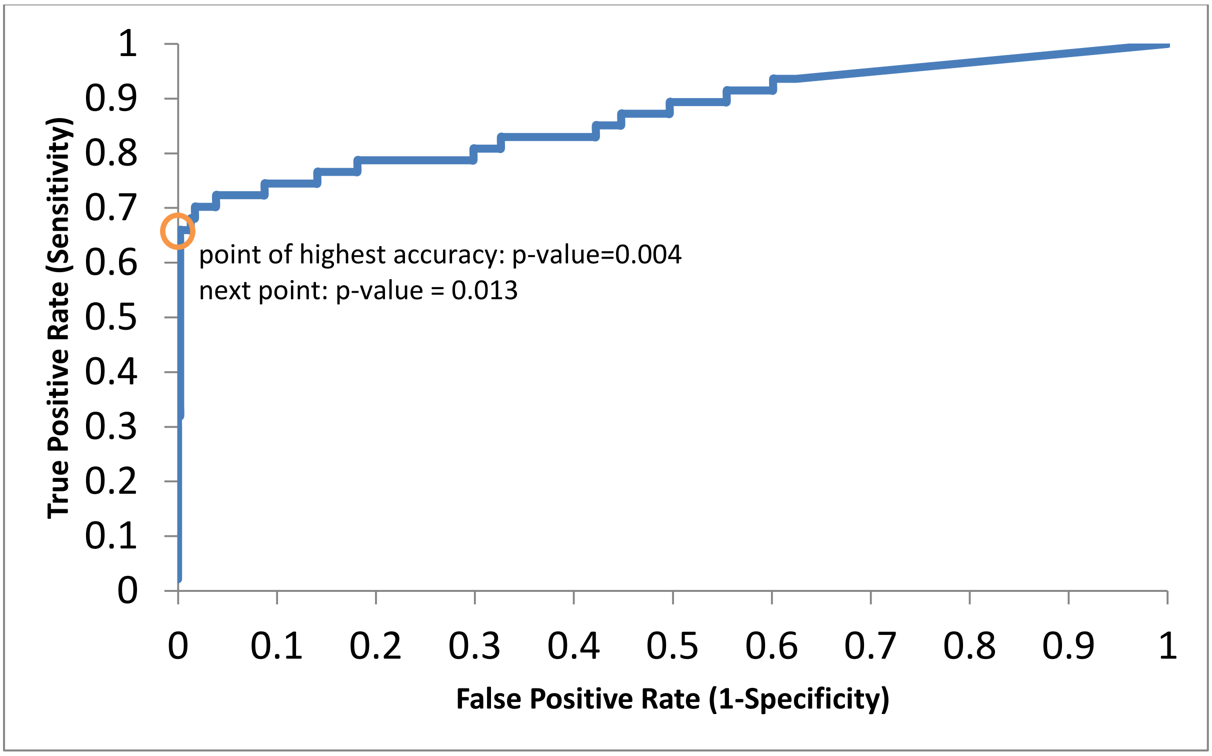

Supplementary Figure S4: Histogram of Pearson correlation coefficients between observed and expected variant frequencies over 326 amplicons in 44 tumour dilution series.

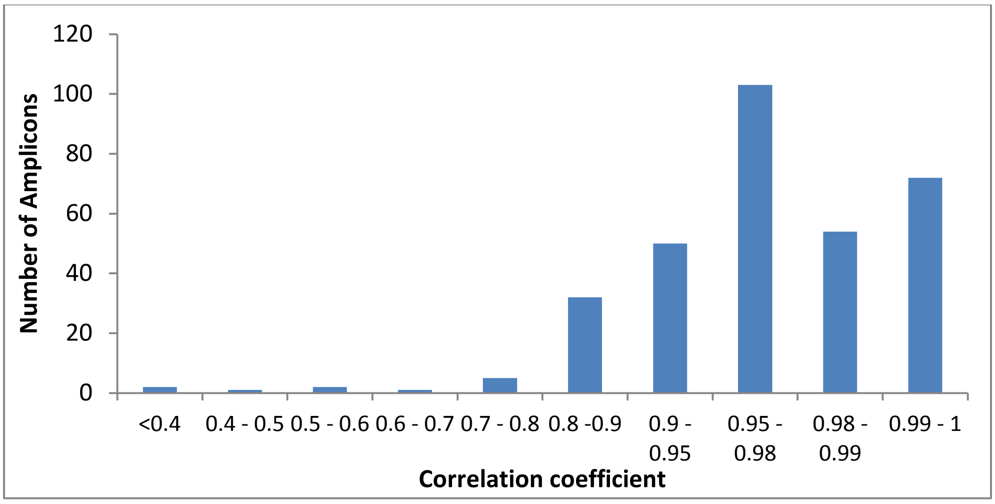

Supplementary Table S1: Patient characteristics including availability of plasma samples and ctDNA detection at selected clinical events.

Patient	Total number of plasma samples	Age	Gender	Stage of primary tumor (T Stage/N stage [no. of lymph nodes involved/no. of lymph nodes harvested]	Site of primary tumor	Pre-primary-surgery plasma available?	ctDNA detected in pre-primary-surgery plasma?	Post-operative (within 1 week) plasma available?	ctDNA detected in post-operative plasma?	Did patient develop recurrence?	Site of recurrence	Plasma samples prior to/at recurrence after primary surgery available?	ctDNA detected pre-recurrence?	Metastectomy / surgery for recurrent disease performed?	Was there recurrence post-metastectomy?	Site of recurrence post-metastectomy	Plasma samples available prior to/at recurrence after metastectomy available?	ctDNA detected pre-recurrence?
417	2	68y	Male	T1N0 [0/8]	Sigmoid Colon	y	n	y	n	n	NA							
441	14	77y	Male	T3N1 [1/11]	Sigmoid Colon	y	n	y	n	n	NA							
494	3	69y	Male	T4aN2 [4/16]	Sigmoid Colon	y	y	y	y	metastatic at diagnosis	NA (metastatic to liver at diagnosis)							
440	2	77y	Male	T3N0 [0/19]	Ascending Colon	y	y	y	n	n	NA							
478	2	64y	Female	T3N0 [0/21]	transverse colon	y	y	y	n	n	NA							
480	2	61y	Male	T3N0 [0/19]	Sigmoid Colon	y	y	y	n	n	NA							
495	2	61y	Male	T3N0 [0/15]	Descending colon	y	y	y	n	n	NA							
130	10	59y	Male	T3N0 [0/12]	Rectum	y	y	y	n	n	NA							
206	9	63y	Male	T3N1c [0/11+ satellite nodule]	Rectum	y	y	y	n	n	NA							
486	4	59y	Male	T4bN0 [0/14]	Sigmoid Colon	y	y	y	n	n	NA							
405	13	62y	Male	T2N1 [2/13]	Rectum	y	y	y	n	y	Lung	y	n	y	y	Liver + Local	y	y
442	1	67y	Female	T3N0 [0/12]	Descending colon	y	y	n		n	NA							
410	1	60y	Female	T2N2 [5/21]	Rectum	y	y	n		n	NA							
76	11	55y	Male	T3N1 [3/21]	Sigmoid Colon					y	Liver	y	y	y	y	Liver	y	y
28	12	66y	Male	T3N2 [4/24]	Rectum					y	Lung and Bone	y	y					
120	23	64y	Female	T4aN2 [9/15]	Rectosigmoid Colon					y	Peritoneum and Lung	y	y					
214	13	66y	Male	T3N0 [0/12]	Transverse Colon					y	Lung, Liver and Peritoneum	y	y					
475	9	56y	Male	T3N1 [2/17]	Rectum					y	Lung	y	y					
30	1	47y	Female	T4aN2 [6/14]	Sigmoid Colon					y	Right and Left Ovary and Tube	y	y					
429	1	60y	Male	T2N0 [0/8]	Rectosigmoid Colon					y	Liver	y	y					
153	11	66y	Male	T3N1 [1/18]	Sigmoid Colon					y	Lung	y	n					
382	1	64y	Female	T3N2 [7/24]	Ascending Colon					y	Brain	y	n					

Supplementary Table 1 (continued): Patient characteristics including availability of plasma samples and ctDNA detection at selected clinical events.

Patient	Total number of plasma samples	Age	Gender	Stage of primary tumor (T Stage/N stage [no. of lymph nodes involved/no. of lymph nodes harvested]	Site of primary tumor	Pre-primary-surgery plasma available?	ctDNA detected in pre-primary-surgery plasma?	Post-operative (within 1 week) plasma available?	ctDNA detected in post-operative plasma?	Did patient develop recurrence?	Site of recurrence	Plasma samples prior to/at recurrence after primary surgery available?	ctDNA detected pre-recurrence?	Metastectomy / surgery for recurrent disease performed?	Was there recurrence post-metastectomy?	Site of recurrence post-metastectomy	Plasma samples available prior to/at recurrence after metastectomy available?	ctDNA detected pre-recurrence?
143	3	59y	Female	T4aN0 [0/15]	Sigmoid Colon					y	Peritoneum , Small bowel and Omentum	n		y	y	Peritoneum	y	y
187	3	72y	Male	T3N0 [0/33]	Sigmoid Colon					y	Anastomosis, para-aortic nodules and peritoneal disease	n		y	y	Peritoneum	y	y
68	1	59y	Male	T2N0 [0/10]	Sigmoid Colon					y	Liver	n		y	y	Liver	y	n
404	1	45y	Male	T3N0 [0/12]	Sigmoid Colon					y	Liver	n		y	y	Liver		
484	4	55y	Female	T3N0 [0/12]	Transverse Colon					y	Liver	n		y	n			
315	4	55y	Male	T4aN0 [0/39]	Rectum					y	lymph node + local recurrence	n		y	n			
398	3	73y	Male	T3N1 [3/21]	Rectum					y	lymph node + local recurrence	n		y	n			
31	2	64y	Male	T3N2 [8/22]	Rectum					y	Lung	n						
38	12	61y	Male	T4aN2 [7/14]	Caecum					y	Liver, LN	n						
51	9	64y	Male	T3N2 [12/21]	Rectosigmoid Colon					y	Lung	n						
260	20	71y	Male	T3N1 [2/17]	Sigmoid Colon					y	Lymph nodes	n						
29	4	45y	Male	T4aN1 [3/13]	Rectosigmoid Colon					y	Liver	n						
183	7	60y	Male	T3N2 [6/24]	Sigmoid Colon					y	Peritoneum and Spleen	n						
198	7	63y	Female	T4aN1 [1/29]	Ascending Colon					y	Ovary	n						
373	4	58y	Female	T3N2 [5/30]	Splenic Flexure					y	Ovary	n						
7	1	73y	Male	T3N1 [1/10]	Rectum					y	Lung	n						
81	6	51y	Male	T3N1 [2/16]	Rectum					y	Lung	n						
18	2	59y	Male	T3N1 [1/13]	Sigmoid Colon					y	Liver	n						
439	13	61y	Male	T4aN2b [10/12]	Rectosigmoid Colon					y	Lymph nodes	n						
61	1	53y	Male	T3N0 [0/15]	Rectum					y	Liver and Lung	n						
188	2	67y	Male	T3N1 [12/46]	Sigmoid Colon					y	Liver	n						
Patient removed from further analysis due to mix-up																		
47	4	54y	Male	T4bN0 [0/12]	Transverse Colon					y	Liver			y	n			
